# Does gene deletion of AMPA GluA1 phenocopy features of schizoaffective disorder?

**DOI:** 10.1016/j.nbd.2010.08.005

**Published:** 2010-08-08

**Authors:** Paul J. Fitzgerald, Chris Barkus, Michael Feyder, Lisa M. Wiedholz, Yi-Chyan Chen, Rose-Marie Karlsson, Rodrigo Machado-Vieira, Carolyn Graybeal, Trevor Sharp, Carlos Zarate, Judith Harvey-White, Jing Du, Rolf Sprengel, Peter Gass, David Bannerman, Andrew Holmes

**Affiliations:** 1 Section on Behavioral Science and Genetics, Laboratory for Integrative Neuroscience, National Institute on Alcohol Abuse and Alcoholism, Bethesda, MD, USA; 2 Department of Experimental Psychology, South Parks Road, University of Oxford, UK; 3 Department of Psychiatry, Tri-Service General Hospital, National Defense Medical Center, Taipei, Taiwan; 4 Laboratory of Molecular Pathology, National Institute of Mental Health, Bethesda, MD USA; 5 Department of Pharmacology, Mansfield Road, University of Oxford, UK; 6 Mood and Anxiety Disorders Research Program, National Institute of Mental Health, Bethesda, MD, USA; 7 Neuroendocrinology Section, Laboratory of Physiologic Studies, National Institute on Alcohol Abuse and Alcoholism, Bethesda, MD, USA; 8 Max-Planck Institute for Medical Research, Heidelberg, Germany; 9 Department of Psychiatry and Psychotherapy, Central Institute of Mental Health, Mannheim, Germany

**Keywords:** glutamate, mouse, stress, anxiety, mania, dopamine, open field test, elevated plus-maze

## Abstract

Glutamatergic dysfunction is strongly implicated in schizophrenia and mood disorders. GluA1 knockout (KO) mice display schizophrenia- and depression-related abnormalities. Here, we asked whether GluA1 KO show mania-related abnormalities. KO were tested for behavior in approach/avoid conflict tests, responses to repeated forced swim exposure, and locomotor responses under stress and after psychostimulant treatment. The effects of rapid dopamine depletion and treatment with lithium or GSK-3β inhibitor on KO locomotor hyperactivity were tested. Results showed that KO exhibited novelty- and stress-induced locomotor hyperactivity, reduced forced swim immobility and alterations in approach/avoid conflict tests. Psychostimulant treatment and dopamine depletion exacerbated KO locomotor hyperactivity. Lithium, but not GSK-3β inhibitor, treatment normalized KO anxiety-related behavior and partially reversed hyperlocomotor behavior, and also reversed elevated prefrontal cortex levels of phospho-MARCKS and phospho-neuromodulin. Collectively, these findings demonstrate mania-related abnormalities in GluA1 KO and, combined with previous findings, suggest this mutant may provide a novel model of features of schizoaffective disorder.

## Introduction

Genes and molecules involved in excitatory synapse function are attractive candidates as risk factors for psychiatric conditions with a broad clinical phenotype such as schizoaffective disorder. Such ‘synaptologies’ have been posited to underlie neurodevelopmental disorders such as autism (Sudhof, 2008) but could also contribute to the pathophysiology of neuropsychiatric disorders with a major developmental component such as schizoaffective disorder. In this context, L-glutamate is the major excitatory neurotransmitter system in the central nervous system and a key regulator of synaptic function and plasticity (Malenka and Bear, 2004). Glutamatergic dysfunction is strongly implicated in schizophrenia and mood disorders (Coyle, 2006).

Glutamatergic neurotransmission is mediated by an array of receptors belonging to the ionotropic (α-amino-3-hydroxy-5-methyl-4isoxazole propionate [AMPAR], *N*-methyl-D-aspartate [NMDAR], kainate) and metabotropic receptor (mGluR) subfamilies. AMPAR are postsynaptic heteromeric proteins composed of one or more glutamate receptor GluA1-GluA4 subunits (Shi et al., 2001). The GluA1/2 heteromer, and coupling to its downstream intracellular signaling pathways, is altered in rodents by treatment with anti-manic drugs (e.g., lithium, valproate) and mania-inducing manipulations (e.g., psychostimulant treatment) (Du et al., 2008; Du et al., 2007). There are also reports of reduced GluA1 gene expression in the post-mortem brains of persons with bipolar disorder (Beneyto et al., 2007).

Important insight into the potential role of the GluA1 subtype has come from studies in mutant mice with targeted deletion of GluA1 (GluA1 ‘knockout,’ KO). For example, we recently reported that GluA1 KO caused multiple behavioral abnormalities considered relevant to schizophrenia (Wiedholz et al., 2008). Interestingly, we and others have also found that GluA1 KO exhibit disturbances on tests for emotionality (Bannerman et al., 2004; Mead et al., 2006; Vekovischeva et al., 2004), and develop a ‘depression-related’ phenotype on various tests that entail repeated exposure to stressful situations (Chourbaji et al., 2008). The pleiotropic nature of genetic deletion of GluA1 suggests that the phenotype of these mutants may more closely model features of more than one neuropsychiatric disorder rather than any one major affective or psychotic condition alone.

There is an increasing view that despite their diagnostic demarcation, certain forms of severe psychotic and mood disorders have a common pathophysiological basis (Owen et al., 2007). This is supported by the observation that the prevalence of schizophrenia and bipolar disorder tends to cluster in families and share a genetic component (Cardno et al., 2002; Kendler et al., 1998; Owen et al., 2007; Park et al., 2004; Potash et al., 2003). It has also recently been shown that bipolar disorder and major depressive disorder share a genetic susceptibility locus (McMahon et al., 2010). Clinically, individuals who exhibit ‘illness during which there is a major depressive, manic, or mixed episode concurrent with symptoms that meet criterion [A] for schizophrenia’ meet diagnostic criteria for schizoaffective disorder (DSM-IV, 1994). However, the specific genetic and molecular factors that put an individual at risk for schizoaffective disorder, rather than a more discrete psychotic or depressive disorder, are not well understood.

Given previous studies have found that GluA1 KO mice exhibit schizophrenia- and depression-related abnormalities, the aim of the present study was to evaluate these mutants for other features of schizoaffective disorder with a focus on the manic component. Mania is characterized by hyperactivity of movement and thought, and can be precipitated or exacerbated by stress (Ambelas, 1979; Malkoff-Schwartz et al., 2000). We assayed GluA1 KO mice for locomotor responses to novelty and stress in an open field and behavioral responses to novel and repeated forced swim. Because the manic component is also associated with increased self-esteem, risk-taking and novelty-seeking, we tested the mutants for anxiety-related and novelty-seeking behaviors in various tasks that differentially test these two drives (elevated plus- and zero-maze, light/dark emergence test, stress-induced hyperthermia).

We went on to explore potential neurobiological mechanisms underlying behavioral abnormalities in GluA1 KO. We tested whether the KO phenotype was rescued by two drugs with known or hypothesized efficacy in bipolar disorder or schizophrenia, lithium and the glycogen synthase kinase-3 beta inhibitor, SB216763. Moreover, given our earlier finding that these KO mice had impaired striatal dopamine clearance (Wiedholz et al., 2008), we tested whether the hyperlocomotor phenotype was rescued by depletion of brain dopamine. To identify molecular mechanisms associated with the GluA1 KO phenotype and its predicted rescue by lithium, we evaluated the activity of two markers (MARCKS and neuromodulin) of protein kinase C (PKC), an intracellular pathway implicated in mania (Szabo et al., 2009).

## Materials and Methods

### Subjects

GluA1 KO were generated as previously described (Zamanillo et al., 1999) on a 129S1/Sv-p^+^Tyr^+^Kitl*^Sl^*^−^*^J^*/+ × 129X1/SvJ background. We have previously found these mice to be normal on a range of simple measures of physical health, neurological and sensory functions (Wiedholz et al., 2008) (Table 1; male WT=8, female WT=6, male KO=8, female KO=6; counts by experimental group (n) in legend), and motor coordination (Palachick et al., 2008). For the overall study, experiments were performed at the NIAAA unless otherwise stated. In order to reduce the number of mice used, but at the same time minimize potential carry-over effects due to, e.g., stress of test exposure, some mice were tested on multiple assays (details given below), with putatively more stressful tests later in a sequence and at least 1 week between tests (unless otherwise stated). Mice were 2-10 months old at the time of the experiments. On the basis of our previous finding that schizophrenia-related phenotypes (Wiedholz et al., 2008), as well fear-related and alcohol-related phenotypes (Feyder et al., 2007; Palachick et al., 2008), in GluA1 KO did not vary as a function of sex, the current study used both males and females in order to increase the power of the analyses, without balancing sex ratio or considering sex as a factor in the analyses. Another one of our studies found similar behavioral results between male and female GluA1 mice across a number of measures (Bannerman et al., 2004).

#### NIAAA experiments

Mice were backcrossed to produce a >75% C57BL/6J background, as verified by genome scan (Wiedholz et al., 2008). WT and KO were littermates bred from heterozygous × heterozygous parents (to reduce potential influence of maternal genotype (Millstein et al., 2006)), either bred at The Jackson Laboratory and transported to NIH at ∼8 weeks of age, or bred in-house. Non-mutant C57BL/6J mice used in the GYKI 52466 experiments were males obtained from The Jackson Laboratory and transported to NIH at ∼8 weeks of age. Mice were housed in same-sex groupings in a temperature- and humidity-controlled vivarium under a 12 h light/dark cycle (lights on 0600 h) and tested in the light phase. Experimental procedures were performed in accordance with the National Institutes of Health Guide for Care and Use of Laboratory Animals and were approved by the local Animal Care and Use Committee. The number of mice used in all experiments is given in the figure legends.

#### Oxford experiments

Mice were maintained on a C57BL/6J × CBA/J background. WT and KO were littermates bred in-house from heterozygous × heterozygous parents. Mice were housed in same-sex groupings in a temperature- and humidity-controlled vivarium under a 12 h light/dark cycle (lights on 0700 h) and tested in the light phase. Experimental procedures were approved by the Home Office.

#### Mannheim experiments

Mice were backcrossed to C57BL/6J for > 10 generations. WT and KO were littermates bred in-house from heterozygous parents. Mice were housed in a temperature- and humidity-controlled vivarium under a 12 h reversed light/dark cycle (lights off 0600 h) and tested in the dark phase. Experimental procedures were approved by the German animal welfare authorities (Regierungspräsidium Karlsruhe).

### Locomotor response to novelty and acute stress

As manic and psychotic episodes can be triggered by stress, we first tested separate cohorts of mice for locomotor responses to 3 different relatively mild stressors [1: a novel open field, 2: acute injection stress (which is sufficient to activate the hypothalamic-pituitary-adrenal axis in mice (Li et al., 2004)), and 3: acute restraint stress], as well as activity in a non-stressful home cage-like environment (Oxford).

#### Novel open field

The open field apparatus was a square arena (39 × 39 × 35 cm) with opaque white Plexiglas walls and floor. It was evenly-illuminated to ∼60 lux. Mice (male WT=8, female WT=7, male KO=8, female KO=7) were placed in a corner and allowed to freely explore for 10 min. Total distance traveled and time spent in the (20 × 20 cm) center was measured using the Ethovision videotracking system (Noldus Information Technology, Leesburg, VA). The effect of genotype was analyzed using Student's t-test.

#### Injection stress

Following a 30-min period of acclimation to the open field (as above), mice (male WT=3, female WT=5, male KO=6, female KO=1) were given an intraperitoneal (i.p.) injection of 0.9% saline (volume of 10 mL/kg body weight) and returned to the open field. Total distance traveled was recorded for a further 30 min. For all i.p. injections, mice were held in a level, supine position by the experimenter, and injected in the abdomen. Injections were made midway between the hip and midline, with the injection needle inserted at ∼30° angle.

#### Brief restraint stress

Following a 30-min period of acclimation to the open field (as above), mice (male WT=7, female WT=3, male KO=4, female KO=5) were removed and restrained in a 50 mL Falcon tube for 5 min before being returned to the open field for 30 min. For both stress experiments, the effects of genotype and time on baseline pre-stress and post-stress locomotor activity were analyzed using 2-factor analysis of variance (ANOVA), with repeated measures for time, followed by Bonferroni-Dunn *post hoc* tests. Stress-induced changes in locomotor activity were analyzed by comparing the minute post-stress with the minute pre-stress (i.e., minute 31 vs. 30).

#### Home cage-like assay (Oxford)

Mice (same as previously tested in the elevated plus-maze; male WT=24, female WT=21, male KO=21, female KO=26) were singly placed in a cage (22.5 × 12.5 × 13 cm) with clean bedding and *ad libitum* food and water for 9.5 hr (09301900 h). Beginning at the onset of the dark phase (1900 h) activity was measured primarily using the Med Associates Threshold system (Med Associates Inc, St. Albans, VT). Threshold pressure pads convert changes in pressure on pads into changes in voltage. The threshold range was set to 20-50V, the time spent between these values being taken as time spent moving. The effect of genotype was analyzed using Student's t-test.

### Response to novel and repeated forced swim stress

We next tested for behavioral responses to the more intense stress of forced swimming. We examined responses to both acute and repeated (2 trials and 5 trials) forced swim test (FST) stress in separate cohorts of mice (apparatus and procedure as described, Hefner and Holmes, 2007). To measure the HPA-axis response to acute swim stress, we assayed blood corticosterone levels 30 min after a single FST (procedure as described, Boyce-Rustay et al., 2007). To analyze behavioral responses to repeated swim stress in greater detail, we conducted a separate experiment (Oxford) using a modified version of the forced swim test that distinguishes immobility, swimming and climbing behavior (Cryan et al., 2005).

#### 2-trial forced swim

We first conducted a 2-trial procedure mimicking the original procedure of Porsolt (Porsolt et al., 1977) in which a 15-min trial 1 is followed by a shorter, 6-min, trial 2 24 hr later. For each trial, mice (male WT=8, female WT=7, male KO=8, female KO=7) were gently placed in a 20 cm-diameter cylinder filled to ∼13 cm with 24 ±1.0°C water (Hefner et al., 2007) and scored for immobility (cessation of limb movements except minor movement necessary to keep the mouse afloat) every 5 sec and expressed as the percent number of instances of immobility during min 3-6 of each trial. This design provides 1) a measure of immobility during min 3-6 of an initial exposure, equivalent to the standard forced swim test procedure used for mice (Cryan and Holmes, 2005), and 2) a measure of the change in immobility produced by a prior lengthy swim exposure. The effects of genotype and trial were analyzed using 2-factor ANOVA, with repeated measures for trial, followed by Bonferroni-Dunn *post hoc* tests.

#### 5-trial forced swim

Here, we examined the development of immobility over an extensive history of fixed-length, repeated swims (in a naïve cohort). Mice (previously tested in the elevated plus-maze and home-cage activity tests; male WT=7, female WT=11, male KO=10, female KO=3) were given a 10-min trial each day for 5 consecutive days. Immobility was scored during min 3-10 of each trial. The effects of genotype and trial were analyzed using 2-factor ANOVA, with repeated measures for trial, followed by Bonferroni-Dunn *post hoc* tests.

#### Corticosterone response to swim

A separate cohort of swim-naïve mice (male WT=3, female WT=6, male KO=6, female KO=6) were exposed to a single 10-min swim stress (as above) and returned to the home cage. Thirty min later, mice were sacrificed (between 1000-1200 h) via rapid cervical dislocation and decapitation to collect trunk blood. Non-stressed controls were sacrificed at the same time. Blood samples were centrifuged at 13,000 rpm for 30 sec. Serum was extracted and assayed for total corticosterone (bound and free) using the Coat-a-Count RIA TKRC1 kit (limit of detection: 5.7 ng/ml; Diagnostic Products Corp, Los Angeles) as previously described (Boyce-Rustay et al., 2007). The effects of genotype and stress were analyzed using 2-factor ANOVA.

#### Modified forced swim test (Oxford)

The apparatus and procedure were the same as for the 2-trial test above with the exception that the cylinder was filled to 15 cm with water and, in addition to immobility, swimming and climbing (movement while in contact with the side of the cylinder in a near-vertical posture) were analyzed during an identical window on both days between minutes 3-6 (male WT=12, female WT=10, male KO=12, female KO=14). The effects of genotype and trial were analyzed using 2-factor ANOVA, with repeated measures for trial, followed by Bonferroni-Dunn *post hoc* tests.

### Approach/avoidance behaviors in tests for anxiety-like behavior

We assessed GluA1 mutants in the elevated plus-maze - a test for anxiety-like behavior based upon a conflict between avoidance and approach (Cryan and Holmes, 2005). Because abnormal mouse phenotypes in the elevated plus-maze appear prone to variation across laboratories (Crabbe et al., 1999), we conducted parallel elevated plus-maze experiments in the NIAAA and Oxford laboratories. In addition, given evidence that different mouse approach/avoidance tasks measure partly distinct forms of behavior (Brigman et al., 2009), we assessed the GluA1 mutants in 2 other approach/avoidance tasks: the elevated zero-maze (Mannheim) and light/dark emergence test (NIAAA). Finally, to further parse anxiety- and novelty-seeking-related phenotypes, we tested for 1) stress-induced hyperthermia, an anxiety assay (Groenink et al., 1994), and 2) responses to repeated exposure to the elevated plus-maze (Holmes et al., 2000).

#### Elevated plus-maze (NIAAA)

The apparatus consisted of 2 open arms (30 × 5 cm; 90 lux) and 2 closed arms (30 × 5 × 15 cm; 20 lux) extending from a 5 × 5 cm central area and elevated 47 cm from the ground (San Diego Instruments, San Diego, CA). The walls were made from black ABS plastic and the floor from white ABS plastic. A 0.5 cm raised lip around the perimeter of the open arms prevented mice from falling off the maze. Mice (previously tested in the novel open field; male WT=7, female WT=7, male KO=8, female KO=7) were placed in the center facing an open arm and allowed to explore the apparatus for 5 min. Time spent in the open arms, center square and closed arms, and entries into the open arms, center square and closed arms was measured by the Ethovision videotracking system (Noldus Information Technology Inc., Leesburg, VA), with the center of mass of the mouse determining its arm location. The effect of genotype was analyzed using Student's t-test.

#### Elevated plus-maze (Oxford)

The apparatus and procedure were the same as the NIAAA version with the following exceptions: 1) the dimensions and lighting conditions were 35 × 6 cm (open arms, 85 lux), 35 × 6 × 20 cm (closed arms, 10 lux) and 6 × 6 cm (center square) and elevated 70 cm from the ground, and 2) the maze was made of dark gray plastic. Behavior was measured by the Ethovision videotracking system, with 3 body points (front, center and back of the body) determining its arm location. The effect of genotype was analyzed using Student's t-test (same mice as tested in the home cage-like assay).

#### Elevated zero-maze (Mannheim)

The apparatus consisted of an annular runway (width 6 cm, outer diameter 46 cm, 50 cm above ground level) comprising 2 enclosed quadrants (10 cm high inner and outer walls) separating 2 open quadrants (25 lux). The walls and floor were made from gray plastic. Mice (all male) were placed in a closed quadrant and allowed to explore the apparatus for 5 min, and behavior was measured by the Ethovision videotracking system, with the center of mass of the mouse determining its arm location. The effect of genotype was analyzed using Student's t-test.

#### Light/dark emergence test

The apparatus comprised a square opaque black Plexiglas ‘shelter’ (16 × 16 × 19 cm) with a single exit (6 × 4 cm) facing out into an open field arena (39 × 39 × 35 cm) with opaque white Plexiglas walls and floor that was evenly-illuminated to ∼90 lux. Mice (previously tested in the novel open field, and elevated plus-maze; male WT=8, female WT=7, male KO=8, female KO=7) were placed in the shelter and allowed to freely explore the whole apparatus for 5 min. Time out of the shelter, number of shelter exits, and time and frequency of scans of the open field from the shelter (head and <4 paws out of the shelter) were manually measured using the Hindsight behavioral observation system (Scientific Programming Services, Wokingham, UK). The effect of genotype was analyzed using Student's t-test.

#### Stress-induced hyperthermia

Mice (previously assayed on the functional observation battery; male WT=3, female WT=3, male KO=7, female KO=0) were individually housed in a clean, empty cage for 5 hr to avoid disturbance resulting from handling of cage mates (Van der Heyden et al., 1997). Core body temperature was measured using a peanut oil-lubricated physiological thermometer probe (Thermalert TH-5, Physitemp, Clifton, NJ) inserted 2 cm into the rectum for ∼10 sec on 2 occasions separated by 10 min. Stress-induced hyperthermia is the increase in core body temperature between the 2 measurements. The effect of genotype was analyzed using Student's t-test.

#### Repeated exposure to elevated plus-maze

Test-naïve mice (male WT=13, female WT=9, male KO=9, female KO=9) were tested as above (NIAAA version) over 2 trials, separated by 24 hr. The effects of genotype and trial were analyzed using 2-factor ANOVA, with repeated measures for trial, followed by Bonferroni-Dunn *post hoc* tests.

### Effects of AMPAR/kainate antagonist GYKI 52466

Behavioral abnormalities in GluA1 KO could arise from either loss of GluA1 function in the adult brain or indirect abnormalities arising from alterations in neural development. One indirect way to address this issue is to ask whether pharmacological inactivation of GluA1 in adult non-mutant mice phenocopies the effects of constitutive GluA1 KO. There are currently no GluA1-selective antagonists, precluding a direct comparison. However, the antagonist GYKI 52466 has relative selectivity for AMPA and kainate receptors over other glutamate receptors and has behavioral effects in rats and NSA mice (Tzschentke and Schmidt, 1997; Vanover, 1998). We tested the effects of systemic GYKI 52466 in non-mutant C57BL/6J mice (genetic background of GluA1 KO; all male) on novel open field and elevated plus-maze assays (in the same mice) under the same conditions which produce a robust phenotype in GluA1 KO.

The novel open field and elevated plus-maze tests used to test C57BL/6J mice involved the same apparatuses and procedures as described above (NIAAA version). For each experiment, mice were tested 30 min after treatment with 0, 0.5, 2.5, 5, or 10 mg/kg GYKI 52466 (Sigma, St. Louis, MO, dissolved in a vehicle of 10% Dimethyl sulfoxide (DMSO) and 90% 0.9% saline) injected i.p. in a volume of 10 mL/kg body weight. This vehicle did not produce any observable behavioral effects. Doses were chosen on the basis of previous behavioral studies in mice (Kapus et al., 2008; Vekovischeva et al., 2007). The effect of dose was analyzed using ANOVA.

GluA1 KO have redistribution of GluA2/3 (Zamanillo et al., 1999), and it remains possible that there is a compensatory functional upregulation of AMPAR. Therefore, we tested whether the effects of GYKI 52466 in the novel open field were altered in KO, as a probe for effects on GluA2/3 in the mutants (male WT=3, female WT=8, male KO=6, female KO=0). Test-naïve mice were injected i.p. with 0 or 10 mg/kg GYKI 52466 and immediately tested in a novel open field for 30 min. A within-subjects Latin square design was employed, in which half the mice received vehicle one day and drug the next day, and vice versa for the other half. The effect of genotype and treatment was analyzed using ANOVA, with repeated measures for treatment.

### Responses to psychostimulants

Prior studies have consistently demonstrated novelty-induced locomotor hyperactivity in GluA1 KO (Bannerman et al., 2004; Chourbaji et al., 2008; Vekovischeva et al., 2004; Wiedholz et al., 2008). This phenotype is potentially relevant to a number of neuropsychiatric conditions (e.g., schizophrenia, bipolar disorder, ADHD), but does not discriminate between them. By contrast, psychostimulants such as amphetamine (Adderall) and methylphenidate (Ritalin) would be predicted to exacerbate a bipolar- or schizophrenia-related phenotype (Anand et al., 2000; Einat and Manji, 2006), but normalize an ADHD-related phenotype given their therapeutic efficacy for ADHD. We therefore tested test-naïve separate cohorts of GluA1 KO for locomotor responses to hyperlocomotor-inducing (sub-stereotypic) doses of amphetamine and methylphenidate.

We tested the hyperlocomotor-inducing effects of amphetamine using a within-subjects experimental design previously used to test for mania-related phenotypes in GluA6 KO mice (Shaltiel et al., 2008). Mice (male and female counts not determined) were given a 60 min period of habituation to the open field and then injected with 2 mg/kg amphetamine (Sigma, St. Louis, MO, dissolved in 0.9% saline and injected i.p. in a volume of 10 mL/kg body weight) and returned to the open field for 10 min. The effect of genotype and (10-min) pre- vs. (10-min) post-drug period was analyzed using ANOVA, with repeated measures for period, followed by Bonferroni-Dunn *post hoc* tests and Bonferroni-corrected t-tests. In addition, the effect of genotype on the drug-induced increase in total distance traveled (=post-drug minus pre-drug) was analyzed using Student's t-test.

We tested the hyperlocomotor-inducing effects of methylphenidate in a separate cohort, using a between-subjects design. Mice (male WT=8, female WT=10, male KO=12, female KO=6) were given a 60 min period of habituation to the open field and then injected with vehicle or 5 mg/kg methylphenidate (Sigma, St. Louis, MO, dissolved in 0.9% saline and injected i.p. in a volume of 10 mL/kg body weight) and returned to the open field for 10 min. Methylphenidate-induced locomotor activity was measured as the difference in drug relative to vehicle locomotor activity. The effect of genotype and drug was analyzed using ANOVA. In addition, the effect of genotype on the drug-induced increase in total distance traveled (=drug minus vehicle) was analyzed using Student's t-test.

### Effects of rapid dopamine depletion

Impaired dopaminergic function is implicated in schizophrenia and bipolar disorder. We previously found that GluA1 KO have impaired dopamine clearance in the striatum, and that the KO hyperlocomotor phenotype was rescued by treatment with the dopamine D2 receptor antagonist haloperidol (Wiedholz et al., 2008). These data suggest that excessive striatal dopamine may underlie the GluA1 KO hyperlocomotor phenotype. To test this hypothesis, we examined the effects of rapid depletion of dopamine (via treatment with the tyrosine hydroxylase inhibitor α-methyl-p-tyrosine methyl ester (AMPT)) on locomotor activity (Costa et al., 2006; Gainetdinov et al., 1999). To verify depletion, striatal dopamine (and serotonin) tissue content, as well as metabolites of these two neurotransmitters, was measured immediately after testing.

Mice (previously tested in stress-induced hyperthermia assay; male WT=16, female WT=15, male KO=14, female KO=10) were injected i.p. (volume of 10 mL/kg body weight) with 0 or 200 mg/kg AMPT (Sigma, St. Louis, MO, dissolved in 0.9% saline) and, 60 min later, tested in a novel open field (apparatus and procedure as described above) for 30 min. Dose was selected based on pilot work and previous studies (Costa et al., 2006; Gainetdinov et al., 1999) to cause significant dopamine depletion but not significant locomotor hypoactivity in WT. The effect of genotype and treatment was analyzed using ANOVA, followed by Bonferroni-Dunn *post hoc* tests.

Immediately after open field testing, a subset of mice (male WT=11, female WT=10, male KO=14, female KO=6) were sacrificed via cervical dislocation and decapitation to verify reduced striatal dopamine content via high performance liquid chromatography (HPLC), as previously described (Boyce-Rustay et al., 2008). A dissection of the striatum (also containing septum) was made on ice and tissue was frozen at -80°C. Frozen samples were homogenized by ultrasonic processing in 800 μL of 0.1 M perchloric acid containing 1% ethanol and 0.02% EDTA and centrifuged for 20 min at 3000 g. Thirty μL of the homogenate was used for analysis using a Luna 5μC18(2), 250 × 2.0 mm column (Phenomenex 00G-4252-B0, Torrance, CA) held at 30°C, Waters Corporation (Milford, MA) 717plus autosampler at 4°C, 510 pump at 0.4 ml/min and amperometric electrochemical detector (EiCOM CB100) set at Eox. 0.82 V. The mobile phase contained 2.8 g 1-heptanesulfonic acid sodium salt, 0.17 g EDTA, 20 ml triethylamine, dissolved in 2.2 L water, pH adjusted to 2.5 with 13 ml 85% phosphoric acid, plus 90 ml acetonitrile. Adjusted to 1 L total volume, these values are: 1.21 g 1-heptanesulfonic acid sodium salt, 0.073 g EDTA, 8.61 ml triethylamine, dissolved in 947.1 ml water, pH adjusted to 2.5 with 5.60 ml 85% phosphoric acid, plus 38.74 ml acetonitrile. The detector output was recorded and analyzed with Waters Empower 2 Chromatography Data Software. No internal standard was used for this assay. The quantification was against external standards injected several times during the run (before the samples, every 12 samples, and post-sample injections). We examined tissue content levels of dopamine and its monoamine metabolites 3,4-dihydroxyphenylacetic acid (DOPAC), homovanillic acid (HVA), and its catechol-*O*-methyltransferase metabolite 3-methoxytyramine (3-MT), as well as norepinephrine (NE), and 5-hydroxytryptamine (5-HT) and its monoamine metabolite 5-hydroxyindole-3-acetic acid (5-HIAA). The effect of genotype and treatment was analyzed using ANOVA, followed by Bonferroni-Dunn *post hoc* tests.

### Effects of GSK-3β inhibitor

There is growing evidence supporting the contribution of glycogen synthase kinase-3 beta (GSK-3β) to the pathophysiology and treatment of schizophrenia and bipolar disorder (Gould and Manji, 2002). We therefore asked whether GSK-3β inhibition would normalize the locomotor hyperactivity and forced swim abnormalities in GluA1 KO by treating mice with 2 mg/kg SB216763 every other day for 11 days (i.e., 6 treatments in total, similar to Mao et al., 2009) and subsequently testing in the open field and repeated FST assays. To mimic the procedure used by Mao et al., we tested mice in both the open field and repeated FST assays.

Test-naïve mice (male WT=12, female WT=14, male KO=11, female KO=15) were treated with 2 mg/kg SB216763 (Sigma, St. Louis, MO, dissolved in 90% 0.9% saline/10% DMSO and injected i.p. in a volume of 10 mL/kg body weight) every other day for 10 days (i.e., as previously described (Mao et al., 2009)). On day 10, mice were tested in the open field for 10 min (apparatus and procedure as described above). Mice received another drug treatment on day 11, and on days 12 and 13 were given a 15 min and then 6-min forced swim exposure, respectively (apparatus and procedure as described above). The effect of genotype and treatment was analyzed using ANOVA.

### Effects of lithium treatment

A key test of whether GluA1 KO behavioral abnormalities are mania-related is whether they are rescued by treatment with an anti-manic drug such as lithium (O'Donnell and Gould, 2007). We chronically treated test-naïve mice with lithium for 14 days (Gould et al., 2007; Shaltiel et al., 2008) and then over subsequent days (while still on-drug) tested in the novel open field (male WT=13, female WT=18, male KO=9, female KO=20) and elevated plus-maze (male WT=6, female WT=12, male KO=6, female KO=10). Because the PKC pathway has been implicated in bipolar disorder and as a target for lithium (Szabo et al., 2009), we probed this pathway at baseline and after lithium treatment by quantifying phosphorylation of the PKC activity markers myristoylated alanine-rich C kinase (MARCKS; male WT=5, female WT=6, male KO=6, female KO=6) and neuromodulin (growth associated protein 43, GAP43; male WT=5, female WT=7, male KO=6, female KO=6). (Note, to avoid potentially confounding effects of stress on these molecular measures, we did not test for lithium effects on forced swim test behavior in this experiment).

Regular rodent chow was loaded with 4 g/kg lithium carbonate (Bio-Serv, Frenchtown, NJ, USA) to produce brain lithium levels of ∼0.55 mM and plasma levels in the human therapeutic range (∼1.0 mM) (Shaltiel et al., 2008). Non-treated controls received the same chow with no lithium. Because lithium produces polyuria and polydipsia, all mice (including controls) had their chow moistened daily in 0.9% saline, and cage bedding was changed approximately three times weekly. On day 15, mice were tested in the open field for 10 min (apparatus and procedure as described above), and the data analyzed in 2-min timebins to investigate within-session effects of lithium. On day 16, mice were tested in the elevated plus-maze (NIAAA; apparatus and procedure as described above). The effect of genotype and treatment was analyzed using ANOVA, followed by Bonferroni-Dunn *post hoc* tests.

On day 17, mice were sacrificed by cervical dislocation and rapid decapitation. With reference to a mouse atlas (Paxinos and Franklin, 2001), the ventral striatum (∼ +1.00 - +0.80 mm AP from Bregma) and ventromedial prefrontal cortex (∼ +1.90 - +1.70 mm AP from Bregma) were dissected by micropunch on ice, flash frozen in liquid nitrogen and stored at -80°C until analysis. Samples were sonicated in Homogenization Buffer A (50 mM Tris-Cl, pH 7.5, containing 2 mM dithiothreitol, 2 mM EDTA, 2 mM EGTA, 50 μM 4-(2-aminoethyl)-benzenesulfonylfluoride hydrochloride, 50 mM KF, 50 nM okadaic acid, 1 mM sodium orthovanidate, 5 mM sodium pyrophosphate, 0.1% NP-40, and 5 μg/ml each of leupeptin, aprotinin, chymostatin, and pepstatin A), spun in the Eppendorf 5810R centrifuge (Westbury, NY) for 20 minutes at 4°C at 20,000 rcf, and the clear homogenate was used as total protein. Aliquots of crude whole-cell homogenates taken from the frontal cortices of mice were used to determine the content of phosphorylated and total protein. Protein concentrations were determined using the Bio-Rad (Hercules, CA) protein assay kit, and the linearity of the protein concentration for immunoblotting was ascertained by resolution of selected concentrations of protein. Equal amounts of proteins were subjected to 10% SDS-PAGE gels and separated by electrophoresis. β-actin was used as an internal control, with no differences observed between genotypes. Proteins were then electrophoretically transferred to nitrocellulose membranes. Nonspecific binding on the nitrocellulose was blocked with Tris Buffered Saline plus Tween 20 (TBST), 10% nonfat dry milk, and then incubated with anti-phospho-GAP43 antibody (Upstate Laboratories, Syracuse, NY), anti-GAP43 antibody (Upstate Laboratories, Syracuse, NY), anti-MARCKS antibody (Calbiochem, Gibbstown, NJ), and anti-Phospho-MARCKS (Ser 152/156) antibody (Cell Signaling Technology, Danvers, MA). The secondary antibodies were horseradish peroxidase-conjugated goat anti-rabbit IgG and goat anti-mouse IgG (Cell Signaling Technology, Danvers, MA). The ECL plus kit (GE Healthcare, Piscataway, NJ) was used as a detection system. Notably, in these experiments, nitrocellulose membranes were first probed with anti-phospho-MARCKS, anti-phospho-GAP43 and then stripped with stripping buffer and re-probed with the anti-MARCKSand anti-GAP43 antibodies. Quantification of the immunoblots was performed by densitometric scanning of the x-ray film using a Kodak Image Station 440 CF and Kodak 1D Image Analysis Software (Eastman Kodak, Rochester, NY). For each gel, the net and sum intensity was determined by the software. Net intensity within each gel was normalized to the averaged gel-value from control-treated WT and the effect of genotype analyzed using Student's t-test.

## Results

### GluA1 KO exhibited locomotor hyperactivity in response to novelty and mild stress

**FIGURE 1:**
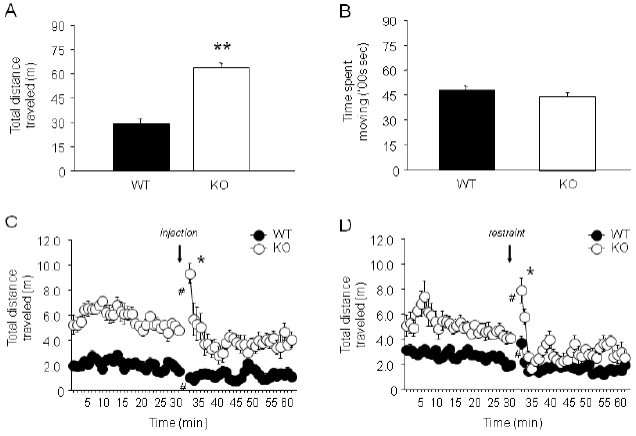
GluA1 KO exhibited locomotor hyperactivity in response to novelty and mild stress (A) KO traveled significantly farther than WT in a 10 min exposure to a novel open field (**p<.01) (n=15/genotype). (B) Movement in a home cage-like environment was not different between genotypes (n=45-47/genotype). (C) KO traveled significantly farther than WT before injection stress. Injection stress produced a transient decrease in locomotor activity in WT but an increase in activity in KO (*p<.05 vs. minute 31 in WT; #*p*<.05 vs. minute 30 in same genotype) (n=7-8/genotype). (D) KO traveled significantly farther than WT before restraint stress. Restraint stress produced a transient increase in locomotor activity in both genotypes, but a greater increase in KO (n=9-10/genotype). Data are Means ±SEM.

KO traveled significantly farther than WT in a novel open field (t=9.16, df=27, *p*<.01) (Figure 1A). By contrast, movement in a home cage-like environment was no different between genotypes (Figure 1B). KO traveled significantly farther than WT before injection stress (main effect of genotype: F1,13=123.92, *p*<.01). Injection stress produced a transient (min 31 vs. min 30) decrease in locomotor activity in WT but an increase in activity in KO (genotype × time interaction: F28,364=6.03, *p*<.01) (Figure 1C). A separate cohort of KO traveled significantly farther than WT before restraint stress (main effect of genotype: F1,16=31.37, *p*<.01). Restraint stress produced a transient (min 31 vs. min 30) increase in locomotor activity in both genotypes, but a greater increase in KO (genotype × time interaction: F29,464=3.41, *p*<.01) (Figure 1D).

### GluA1 KO showed decreased immobility during forced swim

**FIGURE 2:**
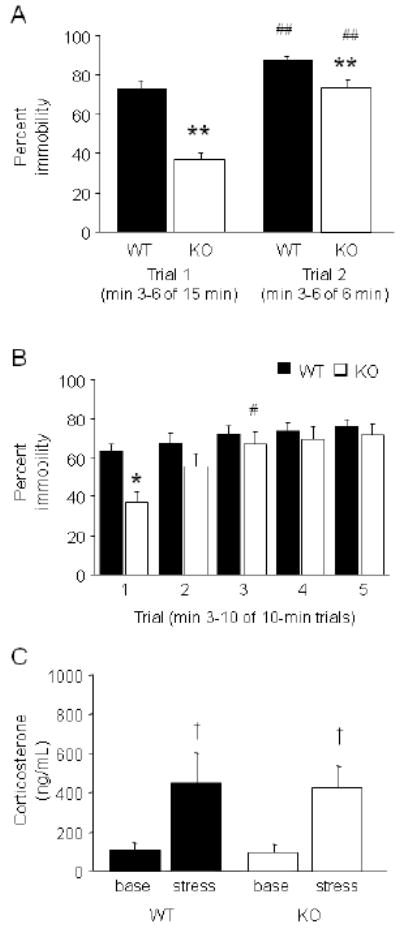
GluA1 KO showed decreased immobility in the forced swim test (A) KO exhibited significantly less immobility than WT over each of 2 FST trials, although the effect was larger during min 3-6 of a 15-min trial 1 than min 3-6 of a 6-min trial 2 (**p<.01, vs. WT/same trial). WT and KO both showed increased immobility from trial 1 to 2 (##*p*<.01 vs. trial 1/same genotype) (n=15/genotype). (B) When tested over 5 × 10 min FST trials, KO displayed less immobility than WT during trial 1 (*p<.05) but not trials 2-5. KO showed a significant increase in immobility by trial 3 (#*p*<.05) (n=13-18/genotype). (C) Serum corticosterone was similarly elevated in WT and KO after a single FST trial, relative to non-stressed baseline (†*p*<.05 vs. base) (n=4-6/genotype/stress). Data are Means ±SEM.

KO exhibited significantly less immobility than WT over each of 2 FST trials, although the magnitude of the difference was greater on trial 1 than trial 2 (genotype × trial interaction: F1,28=21.34, P<.01) and both genotypes showed a significant increase in immobility across trials (Figure 2A). When a separate cohort was tested over 5 × 10-min FST trials, KO displayed significantly less immobility than WT during trial 1 but not trials 2-5 (genotype × trial interaction: F4,116=7.49, *p*<.01) (Figure 2B). *Post hoc* tests revealed that KO, but not WT, showed a significant increase in immobility by trial 3, as compared to trial 1. In a swim-naïve cohort, serum corticosterone was similarly elevated in WT and KO after a single FST trial, relative to non-stressed baseline (main effect of stress: F1,17=12.51, *p*<.01; main effect of genotype: *ns*; stress × genotype interaction: *ns*) (Figure 2C).

### GluA1 KO showed increased swimming in a modified forced swim test

**FIGURE 3:**
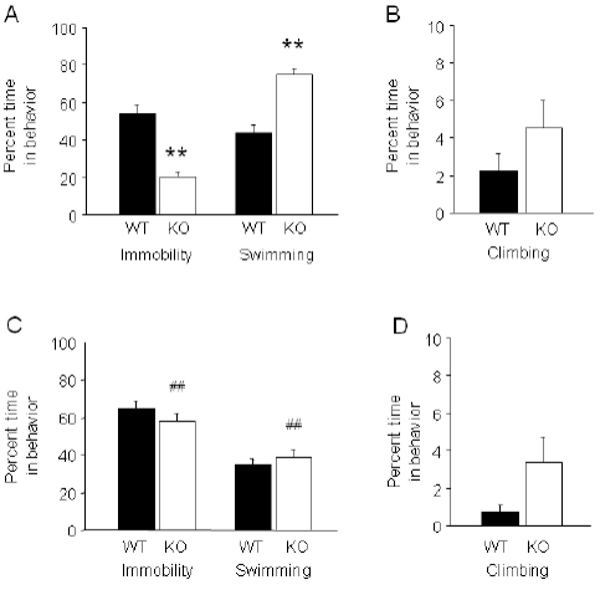
GluA1 KO showed increased swimming in a modified forced swim test (A) KO showed significantly less immobility and more swimming on trial 1 (**p<.01 vs. WT). (B) Climbing did not differ between genotypes on trial 1. (C) KO showed an increase in immobility and a decrease in swimming on trial 2, relative to trial 1, and did not differ from WT in either behavior on this trial (##*p*<.01 vs. trial 1/KO). (D) Climbing did not differ between genotypes on trial 2. n=22-26/genotype. Data are Means ±SEM.

KO showed significantly less immobility (genotype × trial interaction: F1,46=18.98, *p*<.01) and more swimming (genotype × trial interaction: F1,46=21.54, *p*<.01), but no difference in climbing (all effects: *ns*), relative to WT, on trial 1 (Figure 3A,B) but not trial 2 (Figure 3C,D). KO, but not WT, showed a significant increase in immobility and a decrease in swimming from trial 1 to 2.

### GluA1 KO showed increased open arm/quadrant exploration in elevated maze tests

**FIGURE 4:**
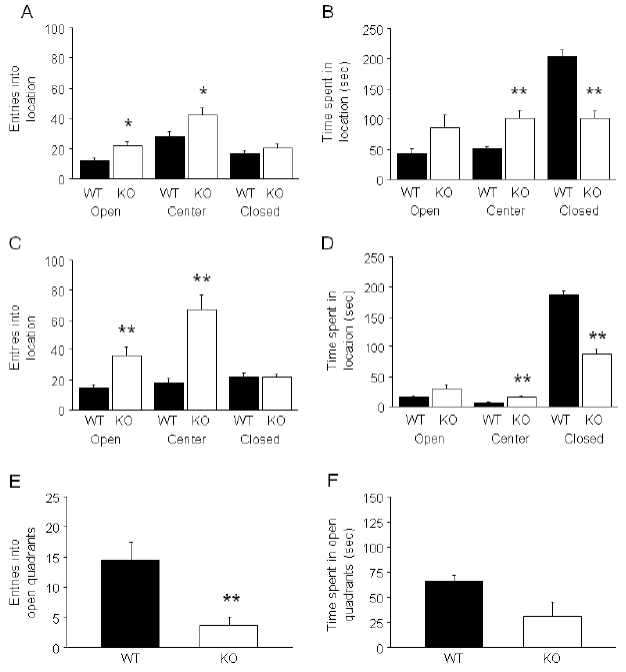
GluA1 KO showed increased open arm/quadrant entries and increased center time in elevated maze tests (A) In the NIAAA elevated plus-maze, KO made more open and center, but not closed, entries than WT (*p<.05 vs. WT) (n=14-15/genotype). (B) KO showed more center time and less closed time, and a trend for more open time, as compared to WT (**p<.01 vs. WT). (C) In the Oxford elevated plus-maze, KO made more open and center, but not closed, entries than WT (**p<.01 vs. WT) (n=45-47/genotype). (D) KO showed more center time and less closed time, and a trend for more open time, as compared to WT (**p<.01 vs. WT). (E) In the elevated zero-maze (Mannheim), KO made fewer open entries (**p<.01) and (F) showed a non-significant trend for less open quadrant time than WT (n=7-8/genotype). Data are Means ±SEM.

In the elevated plus-maze (NIAAA), KO made significantly more open (t=3.10, df=25, *p*<.01) and center (t=2.53, df=25, *p*<.05) but not closed entries than WT (Figure 4A). KO also had significantly more center (t=3.62, df=25, *p*<.01) time and a trend for more open time (t=1.97, df=25, *p*=.0605), but less closed time (t=6.56, df=25, *p*<.01) than WT (Figure 4B).

In the elevated plus-maze (Oxford), KO made significantly more open (t=3.41, df=90, *p*<.01) and center (t=4.63, df=90, *p*<.01) but not closed entries than WT (Figure 4C). KO also had significantly more center (t=2.78, df=90, *p*<.01) time and a trend for more open time (t=1.90, df=90, *p*=.0608), but less closed time (t=10.06, df=90, *p*<.01) than WT (Figure 4D).

In the elevated zero-maze (Mannheim), KO made significantly fewer open entries (t=3.36, df=13, *p*<.01) (Figure 4E) and showed a non-significant trend for less open quadrant time (t=2.10, df=13, *p*=.0554) than WT (Figure 4F).

### GluA1 KO showed increased risk assessment and stress-induced hyperthermia

**FIGURE 5:**
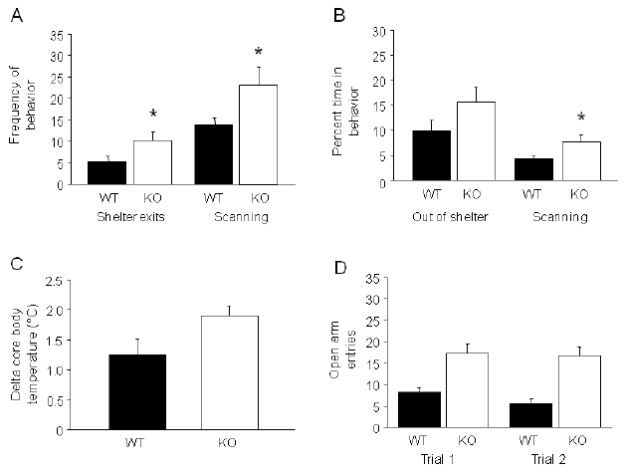
GluA1 KO showed increased exits and risk assessment in the light/dark emergence test and a trend for greater stress-induced hyperthermia (A) In the light/dark emergence test, KO made more shelter exits and showed more frequent scanning than WT (*p<.05 vs. WT) (n=15/genotype). (B) KO did not spend significantly more time out of the shelter than WT, but engaged in more time scanning from the shelter than WT (*p<.05 vs. WT). (C) KO showed a trend for greater stress-induced hyperthermia than WT (n=6-7/genotype). (D) In the elevated plus-maze, KO made significantly more open entries than WT, regardless of trial (n=18-22/genotype). Data are Means ±SEM.

In the light/dark emergence test, KO made significantly more shelter exits (t=2.15, df=26, *p*<.05) and showed more frequent scanning from the shelter (t=2.20, df=26, *p*<.05) than WT (Figure 5A). KO did not spend significantly more time out of the shelter than WT, but engaged in more time scanning than WT (t=2.34, df=26, *p*<.05) (Figure 5B).

KO showed a near significant trend for greater stress-induced hyperthermia than WT (t=2.12, df=11, *p*=.0580) (Figure 5C). Basal temperature was significantly higher in WT than KO (WT=36.7 ±.42, KO=35.7 ±.19; t=2.32, df=11, *p*<.05).

In a naïve cohort tested over 2 exposures to the elevated plus-maze (NIAAA), KO made significantly more open entries than WT, regardless of trial (main effect of genotype: F1,38=24.79, *p*<.01, main effect of trial: *ns*, genotype × trial interaction: *ns*) (Figure 5D). KO also showed significantly more open time than WT regardless of trial (main effect of genotype: F1,38=11.24, *p*<.01; main effect of trial: *ns*; genotype × trial interaction: *ns*) (WT/trial 1 (sec)=15.0±2.4, KO/trial 1=26.1±3.7, WT/trial 2=12.2±3.1, KO/trial 2=30.0±4.7). Closed arm entries were not different between genotypes on trial 1, but were greater in KO than WT on trial 2 (genotype × trial interaction: F1,38=4.94, *p*<.05; WT/trial 1=15.3±1.2, KO/trial 1=15.4±1.5, WT/trial 2=14.3±1.5, KO/trial 2=20.0±2.3).

### GYKI 52466 did not mimic GluA1 locomotor phenotype in C57BL/6J, or alter GluA1 KO phenotype

GYKI 52466 treatment did not significantly alter open field locomotor activity or elevated plus-maze behavior, relative to vehicle, in non-mutant C57BL/6J mice (Table 2). Note, although GYKI 52466 and other AMPAR antagonists exhibit anxiolytic-like effects in rats (Alt et al., 2006; da Cunha et al., 2008; Kapus et al., 2008; Kotlinska and Liljequist, 1998; Matheus and Guimaraes, 1997; Turski et al., 1992), previous work in NMRI mice (light/dark emergence test) (Kapus et al., 2008) and Turku Aggressive mice (social interaction test) (Vekovischeva et al., 2007) found negative (as here) or anxiogenic-like effects.

In KO, GYKI 52466 treatment did not alter the open field locomotor hyperactivity phenotype (main effect of genotype: F1,15=35.33, *p*<.01; main effect of drug: *ns*; genotype × drug interaction: *ns*) (Table 2).

### Psychostimulants exacerbated GluA1 KO locomotor phenotype

**FIGURE 6:**
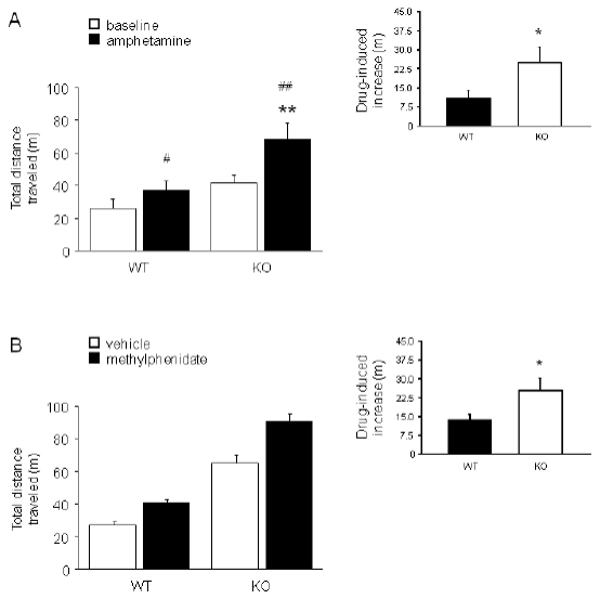
Psychostimulants exacerbated GluA1 locomotor phenotype (A) Amphetamine produced a significant increase in open field total distance traveled in both genotypes (#*p*<.05, ##*p*<.01 vs. baseline). KO given amphetamine traveled significantly farther than WT given this drug (**p<.01). There was a significantly greater drug-induced increase in locomotor activity in KO than WT (inset) (*p<.05) (n=9/genotype). (B) Methylphenidate significantly increased total distance traveled in both genotypes. The drug-induced increase was significantly greater in KO than WT (inset) (*p<.05) (n=9/genotype/treatment). Data are Means ±SEM.

Treatment with amphetamine increased total distance traveled relative to pre-drug baseline in both WT and KO (drug × genotype interaction: F1,17=5.27, *p*<.05; WT drug vs. baseline Bonferroni correct t-test: t=3.24, df=9, *p*<.05; KO drug vs. baseline Bonferroni correct t-test: t=4.17, df=8, *p*<.01) (Figure 6A). However, there was a significantly greater drug-induced increase (relative to pre-drug baseline) in KO than WT (t=2.32, df=16, *p*<.05) (Figure 6A, inset). Total distance traveled was greater in KO than WT after amphetamine treatment, but not during pre-drug baseline activity (probably because this baseline comprised the last 10 min of a 60-min acclimation period, with no injection).

In a separate cohort, treatment with methylphenidate increased total distance traveled relative to vehicle in both WT and KO, and KO traveled farther than WT regardless of treatment (main effect of genotype: F1,32=157.57, *p*<.01; main effect of drug: F1,32=30.96, *p*<.01; genotype × drug interaction: *ns*). However, despite the absence of a genotype × drug interaction, methylphenidate produced a significantly greater increase in locomotor activity in KO than WT when measured as the increase from vehicle (t=2.30, df=17, *p*<.05) (Figure 6B, inset).

### Rapid dopamine depletion paradoxically exacerbated GluA1 KO locomotor phenotype

Vehicle treated KO traveled significantly farther than WT, and AMPT treatment produced an additional significant increase in KO, but had no effect in WT (genotype × treatment interaction: F2,51=7.90, *p*<.01) (Table 3). *Post mortem* striatal tissue levels of monoamines and their metabolites did not differ between genotypes at baseline (all main effects of genotype: *ns*). AMPT treatment significantly decreased striatal dopamine (main effect of treatment: F1,37=24.49, *p*<.01), DOPAC (main effect of treatment: F1,37=81.37, *p*<.01), HVA (main effect of treatment: F1,37=44.31, *p*<.01), and 3-MT (main effect of treatment: F1,37=20.50, p<.01), irrespective of genotype (Table 3). Norepinephrine content was unaffected by treatment or genotype. AMPT treatment significantly increased striatal tissue levels of 5-HT regardless of genotype (main effect of treatment: F1,37=9.27, *p*<.01), but increased 5-HIAA only in KO (genotype × treatment interaction: F1,37=7.29, *p*<.05) (Table 3).

### GSK-3β inhibitor did not rescue GluA1 KO locomotor phenotype

KO traveled significantly farther than WT, regardless of treatment, although there was a borderline genotype × treatment interaction (main effect of genotype: F1,47=33.37, *p*<.01; main effect of treatment: *ns*; genotype × treatment interaction: F1,47=3.55, *p*=.0657) due to a trend for SB216763 treatment to reduce locomotor hyperactivity in KO (Table 4). KO showed significantly less immobility than WT across 2 FST trials, regardless of SB216763 treatment (main effect of genotype: F1,48=21.65, *p*<.01: main effect and interactions with treatment: *ns*) (Table 4). Immobility increased from trial 1 to 2 regardless of genotype or treatment (main effect of trial: F1,48=27.58, *p*<.01).

### Lithium partially rescued GluA1 KO phenotype

**FIGURE 7:**
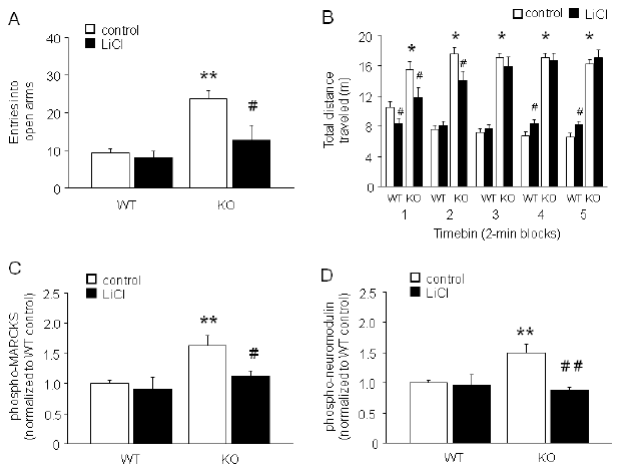
Lithium partially rescued GluA1 locomotor and PKC phenotype (A) In the elevated plus-maze, KO receiving control treatment, but not lithium treatment, made significantly more open entries than WT (n=7-9/genotype/treatment) (**p<.01 vs. WT/control; #*p*<.05 vs. KO/control). (B) In the novel open field, control KO were more active than control WT for all 2-min timebins, and lithium treatment reduced activity during the first timebin in both genotypes such that activity in lithium treated KO was normalized to WT control levels. Lithium treatment also reduced activity in KO, relative to control treated KO, during the second timebin but was ineffective in timebins thereafter. In the fourth and fifth timebins, lithium treatment increased activity in WT. (*p<.01 KO/control vs. WT/control, #*p*<.05 lithium vs. control/same genotype) (n=14-17/genotype/treatment). (C) Levels of phospho-MARCKS in PFC were significantly higher in control-treated but not lithium-treated KO relative to WT (n=5-6/genotype/treatment). (D) Levels of phospho-neuromodulin in PFC were also significantly higher in control-treated but not lithium-treated KO relative to WT (n=6/genotype/treatment). (**p<.01 vs. WT/control; ##*p*<.01, #*p*<.05 vs. KO/control). Data are Means ±SEM.

In the elevated plus-maze, KO receiving control treatment, but not lithium treatment, made significantly more open entries than WT (genotype × treatment interaction: F1,30=5.12, *p*<.05) (Figure 7A) and showed a near significant trend for more percent open time than WT (WT/control=17.0 ±3.0, WT/lithium=14.8 ±3.7, KO/control=48.3 ±5.5, KO/lithium=22.0 ±11.1; genotype × treatment interaction: F1,30=4.06, *p*=.053). Two lithium-treated KO that made no arm entries were excluded from the plus-maze analysis. Closed arm entries was unaffected by genotype or treatment (data not shown).

In the novel open field (Figure 7B), there was a significant main effect of genotype (F1,56=151.83, *p*<.01), as well as a significant interaction between genotype and 2-min timebin (F4,224=18.72, *p*<.01) and treatment and timebin (F4,224=11.07, *p*<.01) for total distance traveled. Post hoc tests showed that whereas control diet KO were more active than control diet WT for all timebins, lithium treatment reduced activity during the first 2-min timebin in both genotypes such that activity in lithium treated KO was normalized to WT control levels. Lithium treatment also reduced activity in KO, relative to control treated KO, during the second 2-min timebin but was ineffective in timebins thereafter. In the fourth and fifth timebins, lithium treatment increased activity in WT. One lithium-treated WT had a seizure at the start of the test, and was excluded from open field analysis. Three other mice (1 control chow-treated KO, 2 lithium-treated WT) were greater or less than 2 standard deviation outliers for total distance traveled for their treatment group, and were excluded from this analysis. Plasma lithium levels measured after the open field were in the therapeutic range in WT (1.05 ±0.07 mEq/L) and KO (1.15 ±0.14 mEq/L). Lithium treatment significantly reduced body weight relative to control treatment (lithium WT weight change relative to pre-drug: -11.8±1.6%; control WT change: +1.8±0.9%; lithium KO: -10.9±2.5%; control KO: +1.9±1.2%) (main effect of treatment: F1,56=64.98, *p*<.01; main effect of genotype: *ns*).

Levels of phospho-MARCKS in prefrontal cortex (PFC) were significantly higher in control-treated (t=3.49, df=10, *p*<.01) but not lithium-treated KO relative to WT (Figure 7C). Levels of phospho-neuromodulin in PFC were also significantly higher in control-treated (t=3.19, df=10, *p*<.01) but not lithium-treated KO relative to WT (Figure 7D). Genotypes did not differ in striatal phospho-MARCKS (WT/control=1.0 ±0.1, WT/lithium=1.2 ±0.4, KO/control=1.4 ±0.3, KO/lithium=1.2 ±0.3) or phospho-neuromodulin (WT/control=1.0 ±0.1, WT/lithium=0.8 ±0.1, KO/control=1.0 ±0.1, KO/lithium=0.9 ±0.1).

## Discussion

The goal of the current study was to test whether mutant mice lacking GluA1 AMPAR subunit exhibit ‘mania-related’ phenotypic abnormalities. Taken together with earlier studies demonstrating that GluA1 KO exhibit schizophrenia- and depression-related phenotypes under certain conditions, the current study suggests that these mice phenocopy features of schizoaffective disorder.

Replicating a phenotype consistently observed in our laboratories and elsewhere (Bannerman et al., 2004; Chourbaji et al., 2008; Vekovischeva et al., 2004; Wiedholz et al., 2008), GluA1 KO showed a marked hyperlocomotor response during exposure to a novel open field. Importantly, this is a specific response to novelty rather than chronic elevation of locomotor activity, because here and previously (Wiedholz et al., 2008), we have shown that GluA1 KO display normal locomotion in the familiar environment of the home cage. Thus, KO appear to be highly sensitive to the stimulatory/provocative characteristics of a novel environment and, indeed, are slow to habituate to such environments (Sanderson et al., 2009). Extending these findings, current data show that open field locomotor hyperactivity can also be provoked in GluA1 KO by exposure to even mild stress. Following a single intraperitoneal saline injection, locomotor activity was suppressed in WT but transiently increased above already elevated baseline levels in GluA1 KO. Similarly, after 5 minute restraint, GluA1 KO again showed an exaggerated short-lasting increase in open field locomotor activity. Collectively, these data show that KO are highly sensitive to environmental provocation, and behaviorally express this response in an open field setting with hyperactivity.

GluA1 KO also showed a strong locomotor activity response (reduced immobility) to the more intense stress of forced swim. As with the open field locomotor hyperactivity phenotype, the forced swim phenotype was highly consistent across different cohorts of mice and across laboratories. Of note, the response diminished with repeated exposure, providing another indication that the hyperactivity response in these mice is particularly strong to novel stimuli. Moreover, a modified version of the test revealed that the reduced immobility was the result of increased swimming behavior but not climbing. Given that reductions in immobility due to increased swimming are produced by antidepressant treatments that boost serotonin, rather than catecholamine, levels (Cryan et al., 2005), this phenotype could reflect a serotonin-related antidepressant-like response (note, basal hippocampal serotonin levels are depressed in GluA1 KO (Chourbaji et al., 2008)). Given our open field data in KO, together with clinical data showing that stress exacerbates psychomotor symptoms in bipolar disorder and schizophrenia (Ambelas, 1979; Malkoff-Schwartz et al., 2000), a more parsimonious interpretation is that the forced swim behavior of these mice is a manifestation of a ‘manic-like’ hyperlocomotor response. Previous studies have similarly interpreted this combination of behavioral abnormalities in other KOs (e.g., Clock KO: Roybal et al., 2007; GluA6 KO: Shaltiel et al., 2008).

Schizophrenia and bipolar disorder are often comorbid with anxiety disorders, although bipolar disorder is more likely to be characterized by risk-taking and novelty-seeking (DSM-IV, 1994). GluA1 KO showed increased entries into the open arms of the elevated plus-maze and light compartment of the light/dark emergence test, as well as consistent trends for increased time spent in these areas. This was another phenotype that was remarkably stable across different laboratories and genetic backgrounds (C57BL/6J and C57BL/6J×CBA/J hybrid), which is particularly notable given evidence that the penetrance of mutant phenotypes in the elevated plus-maze can vary with laboratory (Crabbe et al., 1999) and background (Holmes and Hariri, 2003). Our data are also in general agreement with earlier work showing increased light-compartment exploration and trends for increased open arm time (Vekovischeva et al., 2004). Such profiles are typically interpreted as a decrease in anxiety-like behavior.

However, these anxiety tests are based upon a conflict between threat-related avoidance and novelty-driven approach (Cryan and Holmes, 2005). In this context, data from other tests and previous studies do not fit well with a decrease in anxiety-like behavior in GluA1 KO and indicate a more complex phenotype. KO exhibited a profile in the elevated zero-maze and stress-induced hyperthermia assays more consistent with heightened anxiety-like behavior. It is conceivable that the greater increase in core body temperature in KO could reflect increased muscle activity resulting from stress-induced motor hyperactivity. Nevertheless, the profile in the zero-maze resembles earlier data in the hyponeophagia and black/white alley tests (Bannerman et al., 2004). In addition, KO showed a strong preference for the center of the elevated plus-maze and spent more time scanning the light compartment, behaviors reflective of risk-assessment (Blanchard and Blanchard, 1989; Holmes and Rodgers, 1998). Thus, taken collectively and with our findings in other tests, the profile in the plus-maze may reflect a manic-like increase in approach-behavior perhaps related to heightened ‘novelty-seeking’ or ‘risk-taking.’ Clearly though, additional experiments will be needed to fully understand the nature of this phenotype. For example, it is not clear which facet of certain tests (plus-maze, light/dark) brings out the KO approach-behavior that is not present in others (zero-maze, black/white alley, hyponeophagia) where KO actually show less approach. Of course the distribution of time spent in the different sections of these mazes will, in part, reflect the relative ability of each section to induce exploration which in turn will depend on the salience of the different sections, the starting location of the mouse, and the rate of habituation in the different genotypes (Sanderson et al., 2009), resulting in a complex set of interactions that could differ from one test to the next. Indeed, these data underscore the complexity of these ostensibly simple mouse behavioral tasks that are commonly employed, but not always carefully interpreted (Holmes, 2001).

While the phenotype of locomotor hyperactivity and heightened-approach, at least in certain tests, displayed by GluA1 KO models positive symptoms in schizophrenia and the manic-like component of schizoaffective disorder, it also resembles other neuropsychiatric disorders, including Attention Deficit Hyperactivity Disorder (ADHD) (Brigman et al., in press; Gainetdinov et al., 1999; Powell and Miyakawa, 2006). For example, dopamine transporter (DAT) KO also exhibit novelty-induced locomotor hyperactivity, and this phenotype is normalized by psychostimulants that produce paradoxical calming effects in persons with ADHD (Gainetdinov et al., 1999). A model of bipolar mania or schizophrenia, by contrast, would be expected to be worsened not rescued by psychostimulants, in line with the response shown by schizophrenics and manic persons. Consistent with this, and arguing against an ADHD-like phenotype, acute treatment with the psychostimulant anti-ADHD drugs, amphetamine (Adderall) and methylphenidate (Ritalin), exacerbated rather than reversed open field hyperactivity in KO. One caveat here is these effects were observed over a relatively short, 10 minute, test session, precluding a fuller examination of the time course of genotype differences in psychostimulant responsivity.

Another set of important pharmacological findings was that two weeks of treatment with the anti-manic drug, lithium, normalized the GluA1 phenotype in the elevated plus-maze, although only partially in the novel open field. A similar lithium treatment regimen normalized mania-related behavior in the elevated plus-maze, but not novel open field, in *Clock* mutant mice (Roybal et al., 2007) (but not GluA6 KO, Shaltiel et al., 2008). The relative insensitivity of the open field hyperactivity in GluA1 and *Clock* mutants to lithium treatment may be a reflection of the relatively greater strength of this phenotype. The GSK-3β inhibitor, SB216763, had weak but non-significant reversing effects on the open field but not forced swim hyperactivity phenotype of GluA1 KO. The GSK-3β pathway is a major target of lithium (Gould and Manji, 2005) and SB216763 was recently shown to normalize open field and forced swim hyperactivity in DISC1 mutant mice (Mao et al., 2009). Additional studies are warranted to further probe the GSK-3β pathway in GluA1 KO. However, taken together, the main conclusion from these experiments is that anti-manic drugs rescue at least a subset of the behavioral abnormalities in GluA1 KO. This extends our previous finding that haloperidol, an antipsychotic with efficacy in schizophrenia and mania, also normalizes behavior (in that instance, hyperlocomotion) in GluA1 KO (Wiedholz et al., 2008). This pattern of pharmacological rescue further speaks to a KO phenotype that may have features relevant to both mania and schizophrenia.

In this context, the ability of lithium to rescue phenotypic abnormalities in GluA1 KO was paralleled by normalization of elevated phosphorylation of MARCKS and neuromodulin, two markers of the PKC pathway that interact with GluA1 and are implicated in mania (Szabo et al., 2009). These molecular abnormalities and their reversal by lithium were seen in PFC but not striatum, suggesting the primary PKC alteration was at the cortical level. This raises the possibility that the increased plus-maze approach phenotype in KO is driven by PFC dysfunction (although note that excitotoxic PFC lesions do not typically affect this behavior: Holmes and Wellman, 2009; Rudebeck et al., 2007), while the novelty-induced hyperactivity phenotype may occur independently of frontal regions. It has been suggested that the open field hyperactivity in these mice has a striatal and dopaminergic basis because of our previous finding of reduced striatal dopamine clearance and aforementioned phenotypic rescue by haloperidol (a dopamine D2 receptor antagonist) (Wiedholz et al., 2008). On the other hand, a surprising finding from the current study was that rapid depletion of dopamine via AMPT treatment did not reverse open field hyperactivity in GluA1 KO (as it does in DAT KO: Costa et al., 2006; Gainetdinov et al., 1999), but actually exacerbated the phenotype despite effectively depleting striatal dopamine tissue levels. The reasons for this paradoxical response are unclear and require further study.

The study of constitutive GluA1 KO does not allow us to distinguish between effects of GluA1 absence just during adulthood and consequences arising from developmental disturbances. Of note in this context, a recent microarray analysis revealed multiple changes in the hippocampal expression of calcium signaling proteins in GluA1 KO (Zhou et al., 2009). Although we found that non-mutant C57BL/6J mice systemically treated with the subunit-non-selective competitive AMPAR antagonist, GYKI 52466, did not show KO-like hyperactivity or approach behavior this cannot be readily equated with the complete, subunit-specific nature of the GluA1 KO. Notwithstanding, we cannot exclude the possibility that the mania-related phenotype in GluA1 KO is the consequence of a complex set of changes in glutamate neurotransmission as well as ontogenic insults in, for example, corticostriatal wiring. This would not, however, lessen the potential validity of the mutant as a model of a human affective or psychotic disorder, which are themselves posited to have a major developmental etiological component (Hariri and Holmes, 2006; Harrison and Weinberger, 2005). Another potential issue that was not fully addressed in the current study is the possible modifying influence of sex on the GluA1 phenotype. We had previously found no evidence that male and female GluA1 KO differed on various phenotypic measures, including schizophrenia- and fear related behaviors (Feyder et al., 2007; Wiedholz et al., 2008) and therefore did not design or power the current study to address sex as an interacting factor. We therefore cannot wholly discount a possible sex influence on the current measures, and this remains another interesting question for future studies in these mutants.

In summary, the current study demonstrates that KO of GluA1 produces a profound and selective increase in locomotor activity in response to novelty, and mild and intense stressors. GluA1 KO displayed a complex profile in tests for anxiety-like behavior, with an increased (novelty-seeking or risk-taking related) approach drive in some, but not all, tests. At least some of these behavioral abnormalities were normalized by the anti-manic drug lithium, but were aggravated further by psychostimulants and by rapid dopamine depletion. GluA1 KO and increased approach behaviors were associated with increased PKC activity in PFC. Taken together with previous studies showing these mice exhibit schizophrenia- and depression-related abnormalities, we propose that these mice may phenocopy features of schizoaffective disorder and provide a novel tool for studying the pathophysiology of this disorder.

## Figures and Tables

**TABLE 1:** GluA1 are normal on simple measures of physical health, neurological and sensory function Physical health, sensory reflexes, motor, neurological functions and simple behaviors in an empty cage were generally no different between genotypes. A greater proportion of KO exhibited missing whiskers and bald patches, forepaw clutching and defecation in an empty cage than WT. Unless a unit of measurement is given in parenthesis, data are the percentage of mice within each genotype showing a response (n=14/genotype). Redrawn from (Wiedholz et al., 2008).

	WT	KO
*Physical health*		
Missing whiskers	20	47
Bald patches	13	27
Piloerection	0	0
Exophthalmus	0	0
Palpebral closure	0	0
Straub tail	0	0
Kinked tail	0	0
Kyphosis	0	0
Lordosis	0	0
Body weight (g)	21.5	22.9
Core body temp (°C)	36.5	36.3

*Sensory reflexes*		
Approach response	100	100
Touch response	100	100
Palpebral response	100	80
Tail pink response	100	100
Hotplate paw withdrawal latency (sec)	11.49 ±0.86	9.67 ±0.94
Tail flick latency (sec)	2.02 ±0.07	1.85 ±0.15

*Motor, neurological*		
Splayed limbs	0	0
Forepaw clutch	0	13
Hindpaw clutch	0	0

*Empty cage behaviors*		
Wild running	0	0
Freezing	13	7
Trembling	0	0
Sniffing	100	100
Licking	0	0
Rearing	100	100
Jumping	0	0
Seizure	0	0
Defecation	20	60
Urination	7	13
Head bobbing	0	0
Circling	0	0
Abnormal gait	0	0
Retropulsion	0	7
Prancing forelimbs	0	0

**TABLE 2:** Effects of AMPA/kainate antagonist GYKI 52466 in non-mutant C57BL/6J and GluA1 KO GYKI 52466 did not affect novel open field locomotor activity or elevated plus-maze anxiety-related behavior (n=8-9/dose) in C57BL/6J. GYKI 52466 did not alter the GluA1 KO open field locomotor hyperactivity phenotype (n=6-11/genotype). Data are means ±SEM.

*C57BL/6J*					
	GYKI 52466 dose (mg/kg)				
	0	0.5	2.5	5	10
*Novel open field locomotor activity*					
Total distance traveled (m)	41.9 ±2.0	44.5 ±3.7	49.0 ±2.3	47.6 ±3.3	40.3 ±2.6
*Elevated plus-maze anxiety-related behavior*					
Open arm entries	5.6 ±1.0	5.1 ±1.4	6.1 ±1.1	6.4 ±0.7	7.6 ±1.4
Closed arm entries	22.3 ±1.5	21.4 ±1.6	19.9 ±1.5	20.3 ±1.3	24.6 ±1.5
%Time in open arms	7.4 ±1.8	6.4 ±2.4	9.7 ±2.7	9.8 ±2.2	13.6 ±3.5
%Time in closed arms	58.2 ±1.6	56.3 ±1.8	59.5 ±3.1	59.2 ±2.7	61.6 ±2.9
*GluA1 KO*					
*Locomotor hyperactivity*					
	WT/VEH	WT/GYKI	KO/VEH		KO/GYKI
Total distance traveled (m)	87.7 ±6.8	81.6 ±8.6	168.5 ±12.3		157.2 ±18.2

**TABLE 3:** Rapid dopamine depletion by AMPT paradoxically exacerbated GluA1 locomotor hyperactivity Vehicle treated KO traveled significantly farther than WT, whereas AMPT treatment produced a further increase in KO but had no effect in WT (&&*p*<.01 vs. WT/same treatment; #*p*<.05 vs. KO/vehicle) (n=12-16/genotype/treatment). AMPT treatment decreased striatal tissue levels (ng/g tissue) of dopamine, DOPAC, HVA, and 3-MT, irrespective of genotype (n=10-11/genotype/treatment) (**p<0.01). AMPT treatment significantly increased striatal serotonin regardless of genotype (††p<0.01), but increased 5-HIAA only in KO (#*p*<.05 vs. KO/vehicle). Norepinephrine content was unaffected by treatment or genotype. Data are Means ±SEM.

	WT		KO	
	VEH	AMPT	VEH	AMPT
*Novel open field*				
Total distance traveled (m)	61.6 ±5.9	49.2 ±4.2	115.2 ±11.7&&	155.9±15.4&&#
*Striatal monoamine levels*				
Dopamine**	3555.8 ±599.1	937.5 ±281.9	2797.6 ±461.5	1235.1 ±285.8
DOPAC**	344.7 ±32.9	80.8 ±15.3	270.8 ±33.9	84.8 ±10.1
HVA**	649.6 ±76.6	196.2 ±26.4	561.4 ±79.4	241.3 ±34.3
3-MT**	123.8 ±17.1	31.9 ±10.7	93.2 ±18.1	50.2 ±13.2
Serotonin††	1080.6 ±46.9	1202.3 ±65.3	1054.7 ±64.9	1343.2 ±86.4
5-HIAA	657.8 ±49.2	557.7 ±48.3	540.8 ±41.5	720.8 ±65.9#
Norepinephrine	565.6 ±55.0	569.5 ±55.2	685.4 ±58.6	578.9 ±48.6

**TABLE 4:** GSK-3β inhibitor did not rescue GluA1 locomotor phenotype KO traveled significantly farther than WT, regardless of treatment, although there was a trend for SB216763 treatment to reduce locomotor hyperactivity in KO (n=13/genotype/treatment). KO showed significantly less immobility than WT across 2 FST trials, regardless of SB216763 treatment. Immobility (min 3-6) increased from trial 1 to 2 regardless of genotype or treatment. (n=13/genotype/treatment; same mice as for novel open field test). Data are Means ±SEM.

	WT		KO	
	VEH	SB216763	VEH	SB216763
*Novel open field*				
Total distance traveled (m)	36.4 ±4.7	34.8 ±6.1	71.7 ±9.1	55.6 ±3.4
*Forced swim test*				
Trial 1 percent immobility	34.1 ±7.0	39.4 ±6.4	12.7 ±3.0	12.5 ±3.5
Trial 2 percent immobility	49.7 ±7.5	62.7 ±6.9	30.6 ±7.6	35.1 ±7.2
